# Provision of exercise services in patients with peripheral artery disease in the United Kingdom

**DOI:** 10.1177/17085381211035259

**Published:** 2021-08-04

**Authors:** Amy E Harwood, Sean Pymer, Said Ibeggazene, Lee Ingle, Eddie Caldow, Stefan T Birkett

**Affiliations:** 1 Centre for Sport, Exercise and Life Sciences, Faculty of Health and Life Sciences, Science and Health Building, Whitefriars Street, RinggoldID:120958 Coventry University, Coventry, UK; 2 Academic Vascular Surgical Unit, 12195 Hull York Medical School, Hull, UK; 3 College of Health, Wellbeing and Life Sciences, 7314 Sheffield Hallam University, Sheffield, UK; 4 Department of Sport, Health and Exercise Sciences, Faculty of Health Sciences, 4019 University of Hull, Hull, UK; 5 School of Health and Society, 7046 University of Salford, Salford, UK; 6 School of Sport and Health Sciences, 6723 University of Central Lancashire, Preston, UK

**Keywords:** Peripheral artery disease, intermittent claudication, survey, best practice

## Abstract

**Objectives:**

Supervised exercise programmes (SEPs) are a vital treatment for people with intermittent claudication, leading improvements in walking distance and quality of life and are recommended in multiple national and international guidelines. We aimed to evaluate the use and structure of SEPs in the United Kingdom (UK).

**Design:**

We conducted an anonymous online survey using the Jisc platform comprising of 40 questions. The survey was designed to address key areas such as access, provision, uptake and delivery of SEPs in the United Kingdom. Ethical approval was obtained from Coventry University (P108729).

**Methods:**

The list of trusts providing vascular services was obtained from the National Vascular Registry (NVR) report. The survey was disseminated via social media, The Vascular Society of Great Britain and Ireland and the Society for Vascular Technology. Data were exported to a Microsoft Excel document and analysed using simple descriptive statistics.

**Results:**

Of 93 vascular units identified, we received response from 48. Of these, 23 had access to an exercise programme (48%). The majority of SEPs were exclusively for PAD patients (77%), with 21% using integrated services. 67% of respondents were providing a circuit-based programme, and 5 out of 23 were meeting the dose recommendations in the UK National Institute for Health and Care Excellence (NICE) guidelines. Respondents felt that programmes were moderately to extremely important to patients, slightly to very important to clinicians and not at all important to slightly important to commissioning/funding bodies.

**Conclusion:**

SEPs are a well-established first-line treatment for patients with IC and they are recommended by NICE guidelines. Despite this, many patients still do not have access to an exercise programme, and clinicians do not feel that they have support from commissioning/funding bodies to develop them. There is an urgent need for funding, development and delivery of SEPs in the United Kingdom.

## What this paper adds

We have provided a comprehensive overview of the availability, structure and delivery of supervised exercise programmes in the United Kingdom. We have also identified barriers to implementation and characterised how clinicians feel that programmes are valued by patients and commissioning bodies. There is an urgent need for funding, development and delivery of SEPs in the UK.

## Introduction

Globally, over 236 million people are estimated to have peripheral artery disease (PAD).^2^ A classic symptom of PAD is intermittent claudication (IC), which is characterised by muscle pain or discomfort in the legs brought on by physical exertion.^3^ This pain can be severely disabling and is associated with reduced walking duration, functional capacity, balance and muscle strength.^4-6^

Across many national and international clinical guidelines, the first-line treatment of IC includes secondary prevention of cardiovascular disease risk factors and a supervised exercise programme (SEP).^7-10^ These SEPs generally include a walking-based programme to moderate or maximal claudication pain, typically 3 days per week for around 60 min.^11^ SEPs are efficacious for improving clinical indicators such as maximum walking distance and quality of life.^7,12^ However, it is acknowledged that the overall availability of programmes ^13,14^ and uptake and adherence^15^ to programmes is low. Further, the adherence to guideline recommended therapy is low^16^ Indeed, a survey we conducted in 2016 demonstrated that only 39% of vascular units in the United Kingdom had access to a SEP.^13^ This lack of availability was also recently highlighted in the United States, whereby 54% of respondents did not have access to a programme.^17^ This information should have raised awareness of this limited availability and led to increased SEP provision. For centres with a SEP, the lack of detail and consistency between guidelines may impact upon effective implementation.^11^

We therefore aimed to evaluate the availability, use and structure of SEPs in the UK *National Health Service (NHS)* and update our previous survey from 2016. We wanted to understand how programmes are being implemented by whom and where and how respondents thought exercise programmes are valued by patients, clinicians and commissioning/funding groups. We also wanted to identify possible barriers to implementation.

## Methods

### Study design

We developed an English language anonymous online survey using the Jisc online survey platform (https://www.onlinesurveys.ac.uk/). The survey consisted of 40 questions, although not all questions were presented to all respondents as Boolean operators were used to determine whether further relevant questions were asked according to how questions were answered. The survey was designed to address key areas such as access, provision, uptake and delivery of SEPs in the United Kingdom. Most questions were closed, but there was also the option to provide comments and/or responses to some questions where an ‘other’ option was selected. The full survey outline is provided in the supplementary material.

Primary ethical approval was obtained from the Research Ethics Committee at Coventry University (P108729) prior to the commencement of the survey. Respondents provided electronic consent via a tick box at the beginning of the survey and could not access the survey if they did not consent to participate.

To ensure clarity, appropriateness and functionality, we developed the survey in conjunction with vascular consultants, nurses, physiotherapists and exercise physiologists and piloted it prior to disseminating it more widely.

### Study respondents

We aimed to obtain responses from all trusts who carry out vascular clinics and operations for PAD. The list of trusts providing vascular services was obtained from the National Vascular Registry (NVR) report,^1^ with a total of 93 trusts identified. First, the survey was disseminated via social media (Twitter^TM^), The Vascular Society of Great Britain and Ireland news page and the Society for Vascular Technology. Emails were then sent to trusts who had not responded. Respondents to the survey were asked to identify which trust they worked for and what role they undertook, but no other personal identifying questions were asked to maintain anonymity.

### Data analysis

Data were exported from the Jisc online survey platform to a Microsoft Excel document and analysed using simple descriptive statistics. No responses were excluded from the analysis.

## Results

We identified 93 hospital trusts in the United Kingdom with dedicated vascular units from the most recent NVR database report. Of these, we received responses from 48 units (52%). In addition, we had 12 responses from additional healthcare trusts and one GP practice response. We also had two responses from the same trust but covering separate hospital/spoke sites. Therefore, in total, we had 63 respondents. The survey was completed by a variety of people including nurses, vascular consultants and registrars, podiatrists, exercise professionals and physiotherapists. Overall, 48% of respondents had access to a SEP, 49% did not have access and 3% did not know. Specifically, 23 vascular units indicated that they had an exercise programme.

### Service model

**Figure 1.**
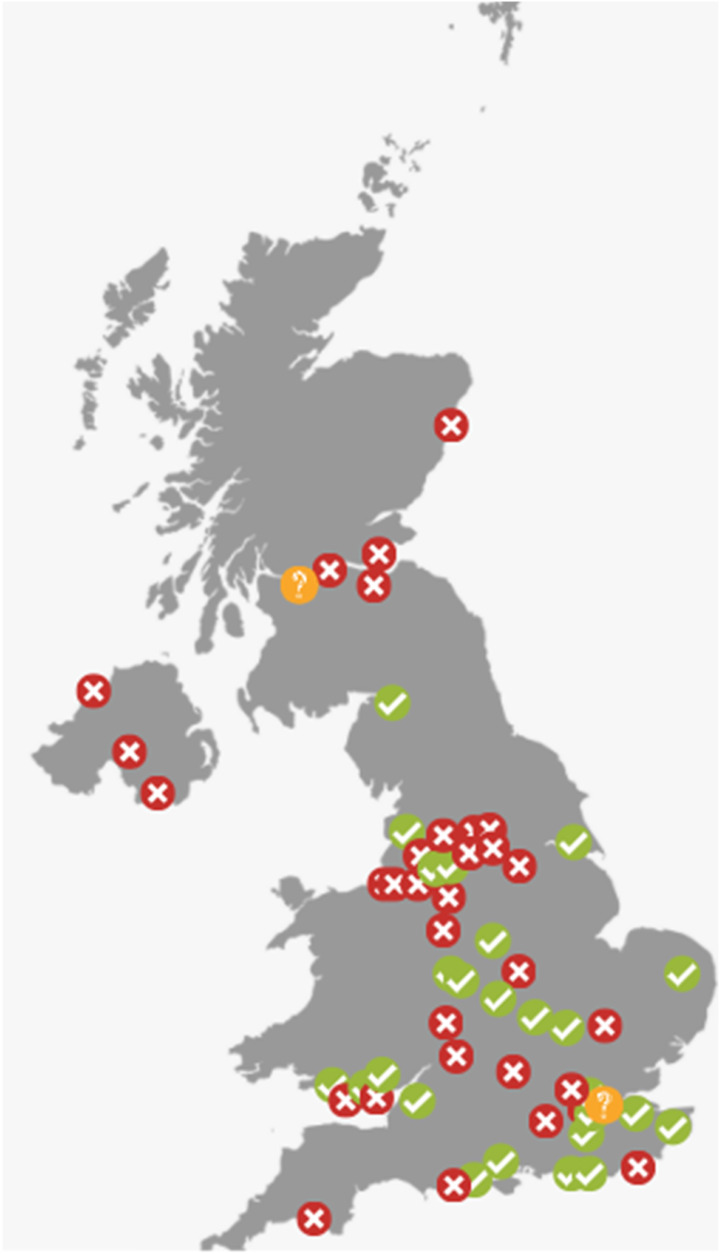
Overview of access to supervised exercise programmes (tick = access, cross = no access and question mark = don’t know).

Figure 1 shows the limited access to SEPs across the United Kingdom and the spread of services. The majority of SEPs were hospital-based (62%) with community (31%) and spoke services (7%) also providing programmes. Most were NHS funded (77%), while 10% did not know how their programme was funded. Nurses were most often the clinical lead for the SEP (42%), followed by physiotherapists (21%), vascular consultants (17%), exercise professionals (10%) and a joint nurse and physiotherapist lead (3%). Some centres (7%) did not know or did not have a clinical lead. Most SEPs provided some form of formal education (76%), however, 17% did not, while 7% did not know. Patients were provided with recommendations for increasing their habitual physical activity in most programmes (80%). Home-based exercise booklets and pedometers were the most adopted methods to use at home. An assessment of patients’ cardiovascular risk factors prior to entering an exercise programme was undertaken by 67% of respondents, with 7% not undertaking any form of risk factor assessment prior to entry and the remaining stating that risk factors were assessed by a vascular consultant/registrar prior to referral.

### Programme delivery

The majority of SEPs were exclusively for PAD patients (77%) although some integrated PAD into cardiac rehabilitation (14%) and multi-morbidity (9%) programmes. The sessions were mostly group-based (73%) and predominantly led by a physiotherapist (36%), followed by an exercise professional (25%), a nurse (21%) or other (17.9%). Table 1 one provides an overview of the exercise testing and prescription (dose) as reported by respondents. The most commonly cited patient barrier to a SEP was time, both in terms of personal time or travel time. Other barriers included no transport or an inability to access public transport, financial limitations and too much pain when walking.

**Table 1.** Overview of programme delivery in supervised exercise programmes.

	Responses % (n)	Description
Conducted baseline testing	Yes – 80% (24/30)	Baseline tests included ABPI and/or a form of walking exercise test
	No – 10% – (3/30)	
	Don’t know – 10% (3/30)	
Pre-programme exercise test	Yes – 83% (20/24)	Exercise tests included a graded or constant load treadmill test, the ISWT or the 6MWT. Strength was measured in two programmes
	No – 17% (4/24)	
Methods for prescription	Claudication pain scale – 50% (13/26)	The claudication pain scale (0–4 or 1–5). No prescription included patients who ‘self-prescribed’ during the exercise sessions. Mixed relates to the use of RPE and %HRR in conjunction with the pain scale
	No prescription – 38% (10/26)	
	Mixed – 12% (3/26)	
Programme duration (Weeks)	12 weeks – 50% (15/30)	
	<12 weeks – 23% (7/30	
	>12 weeks – 13.5% (4/30)>12 weeks – 13.5% (4/30)	
	Don’t know – 13.5% (4/30)	
Session frequency (Days)	1x week – 50% (15/30)	
	2x week – 23% (7/30)	
	3x week – 4% (1/30)	
	Don’t know/other – 23% (7/30)	
Session duration (Minutes)	30 mins–7% (2/30)	Average session duration of programmes was 1226 min over 12 weeks, with 5 out of 23 meeting the NICE recommendation of 2 h per week for 12 weeks (1440 min)
	30–60 mins – 70% (21/30)	
	>60 mins – 7% (2/30)	
	Don’t know – 16% (5/30)	
Mode of exercise	Walking only – 13% (4/30)	Mixed included walking in conjunction with a circuit format and/or RT. Only 30% of programmes included a RT component in the exercise sessions
	Mixed – 67% (20/30)	
	Don’t know – 20% (6/30)	
Post-programme exercise test	Yes – (66%) (20/30)	Exercise tests included a graded or constant load treadmill test, the ISWT or the 6MWT.
	No – 17% (5/30)	
	Don’t know - 17% (5/30)	
		Strength was measured in two programmes

^a^ABPI = ankle brachial pressure index; ISWT = incremental shuttle walk test; 6MWT = six-minute walk test; RPE = rating of perceived exertion; %HRR = percentage of heart rate reserve; RT = resistance training.

### Requirement and perceptions of service provision

**Figure 2.**
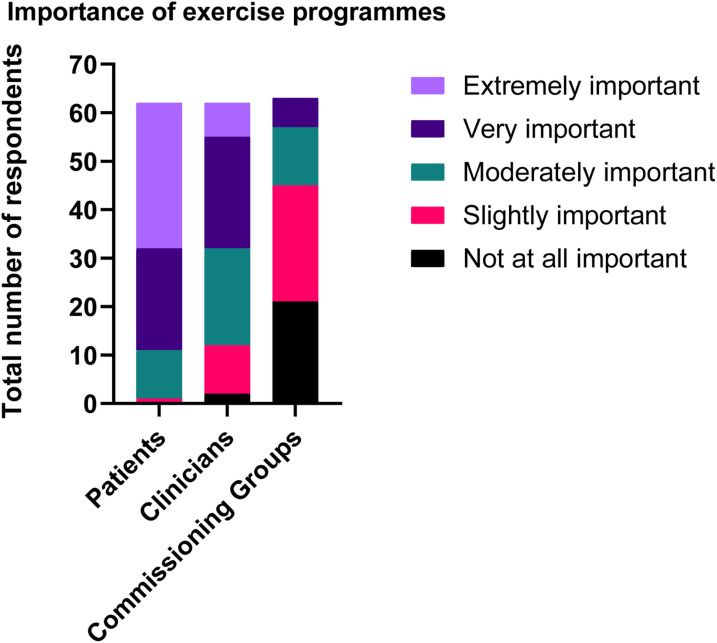
Overall respondents’ views on importance of supervised exercise programmes to patients, clinicians and commissioning groups.

The resources used to guide SEPs included national guidance such as the National Institute for Health and Care Excellence (NICE), research articles, The Circulation Foundation website and/or sport and exercise science statements. Word of mouth and expert opinion were also reported. Clinicians reported that access to exercise facilities, additional staff with expertise, equipment and funding would help with the implementation of an exercise programme where one was not available. If centres did not have a specific exercise programme, basic walking advice and smoking cessation was often provided to patients. Only 30% of programmes offered a specific home-based exercise prescription which included a home-based exercise booklet or structured exercise guidance. Figure 2 demonstrates that most respondents felt that programmes were moderately to extremely important to patients, slightly to very important to clinicians and not at all important to slightly important to commissioning and funding bodies.

## Discussion

SEPs are recommended by vascular guidelines and clinical groups as the first-line treatment for patients with IC. Particularly within the United Kingdom, NICE has advocated SEPs since 2012.^8^ However, this survey highlights that access to SEPs still remains highly variable across the United Kingdom with limited change since surveys conducted in 2009 and 2016.^13,18^ It is important to consider that some centres that did not respond to this survey may offer a SEP, meaning that the figure of 48% may be a slight underestimation. Nevertheless, Figure 1 clearly demonstrates that there is a lack of service provision across large areas of the country, indicating that some patient may not be receiving the optimal treatment. It also demonstrates that some patients may need to travel a considerable distance depending on the area covered by each trust; indeed, travel and financial limitations were reported as barriers to SEPs in this survey. However, we acknowledge the possibility of missing data in regions like the North East.

One interesting finding is that while programmes are deemed to be extremely important to clinicians and clinicians feel that they are valuable for patients, they are considered less important to commissioning/funding bodies who are the ones responsible for deciding if a service needs implementing within the hospital setting. Indeed, funding (as with the last survey in 2016) was highlighted as a major barrier to implementation.^13^ This is despite the recommendations made by NICE. Further investment from commissioning groups for SEPs is desperately needed and this requires urgent attention.

### Integration into existing service

One way of mitigating the need for new exercise programmes could be via the integration of patients with IC into existing infrastructure such as cardiac rehabilitation.^19^ Indeed, we had three respondents indicating that their PAD class was run in conjunction with cardiac rehabilitation with two of these as part of a multi-morbidity rehabilitation model. The coronary artery disease patient and the PAD patient have a shared atherosclerotic pathophysiology and often have similar risk factor profiles and common comorbidities (hypertension, diabetes and pulmonary disease). Cardiac rehabilitation is well developed in the United Kingdom, with 233 programmes available,^20^ providing a potential opportunity to integrate services where they are currently not available. There is limited evidence for integrated rehabilitation into cardiac services or community schemes, so it remains to be established whether outcomes for patients are the same irrespective of the rehabilitation service.^21^ If services are integrated, providers will need to ensure familiarity with various vascular-specific outcome measures (such as maximum walking distance) and the requirement to incorporate exercises that provoke claudication pain, to align with recommended exercise prescriptions.^11^

### Clinical guidelines for exercise services

Alongside funding restrictions and availability of facilities, another aspect that may limit SEP development is the lack of detail provided in the NICE guidelines to support implementation. The simple description of ‘2 hours of supervised exercise a week for a 3-month period encouraging people to exercise to the point of maximal pain’ does not provide enough detail to fully allow for easy implementation. Indeed, the exercise prescription differed vastly between centres in our survey, likely due to this limited guidance. We recently published a more comprehensive summary of exercise prescriptions to aid clinicians implementing exercise programmes.^11^ Outlined components included a clinical assessment, risk stratification and pre-exercise (baseline) testing to establish an appropriate exercise dose (intensity and time). This baseline testing should have at least one method of measuring walking distance (graded exercise test or six-minute walking distance conforming to appropriate guidelines).^22,23^ This measurement should also be repeated or continuously evaluated to ensure that the training intensity is sufficient. During the exercise session, a validated scale such as the claudication pain scale should be used to ensure that patients are walking to maximal pain where tolerated.^24^ Based on the available literature, the exercise programme should ideally be provided at least 3 days per week for at least 3 months, up to 60 min per session.^7^ It is encouraging that many of the respondent’s programmes followed these guidelines, although most programmes were still conducted less than three times per week which may be suboptimal.^25^ By providing such detailed guidance, we may have made implementation easier, while also addressing some of the knowledge-based concerns that were highlighted by respondents who did not have an exercise programme, potentially reducing some provision barriers.^11^

### Exercise modality

Interestingly, while evidence in the literature indicates that walking should be the primary mode of exercise, which is replicated in the national and international guidance^11^ most of our respondents who had exercise programmes were providing a circuit-based programme format. It is possible that equipment limitations (i.e. treadmills), most likely make treadmill-based SEPs relatively infeasible in clinical practice, due to cost and limits on number of people per session. Furthermore, solely walking-based interventions may not be the most preferred option for patients, and a circuit-based format provides the opportunity to incorporate resistance/strength training exercises, which will not only be beneficial for muscle strength but also walking distance.^26,27^

### Uptake and adherence

Encouragingly where data were available from sites, approximately 80–95% of referred patients started the exercise programme, with around 50% of these completing it. A number of respondents were unaware of patient attendance rates, and so these findings provide a limited understanding of real-world uptake and adherence rates, although we know that generally uptake and adherence rates are much lower than this.^15^ To support SEPs and rehabilitation services for patients with IC, to record uptake and adherence and to ensure quality and effectiveness of delivery, a model based on the ‘National Audit of Cardiac Rehabilitation’ could be considered to record vital service-level information. Presently service evaluation for exercise programmes in PAD is lacking and there is no standardised framework to support it. Alongside this, the development of standardised pre- and post-exercise evaluation principles is important to demonstrate improvements in patients and to evaluate different services and their differing prescriptions. Not all respondents in the survey indicating that their centre were able to conduct exercise assessments, particularly post-exercise evaluations.

### Alternative modes of provision

While we know that SEPs are the ‘gold-standard’ method of exercise delivery,^28^ several programmes reported the use of home-based exercise prescription, in particular the use of an exercise booklet. While these programmes are not currently recommended by NICE, they have been important during the COVID-19 pandemic and have some evidence to support their use. Various models of home-based exercise programmes have been evaluated.^29,30^ However, evidence is heterogenous largely down to variations in the methods of delivery, components of the programme and variability in the exercise dose. A recent systematic review has demonstrated that while home-based programmes are inferior to SEPs, they are certainly better than providing no exercise or basic exercise advice.^30^ Furthermore, including a form of active monitoring such as a pedometer or other device increases the benefit of a home-based programme.^30^ Monitoring also means that patients are provided with regular feedback and that the exercise prescription can be structured and personalised. There is, however, a need to further develop and evaluate home-based exercise interventions, particularly those including smart technology such as app-based platforms which can provide feedback and opportunities for remote supervision. It is crucial that patients are part of this development in a co-production format, so that they are designed to meet the needs of the end-user. We also need to understand and trial the delivery of home-based exercise services to understand what barriers there may be. Ultimately it may not be a one-size-fits-all approach with a need to have several different options for patients.

### Limitations

Despite our best efforts to obtain a response from every UK vascular unit registered on the most recent NVR report, we did not capture all data. However, our response rate is similar to that of 2016, and we believe that our findings are representative of all centres. We also anticipated a response bias in favour of those who had exercise programmes. However, the largest number of respondents was those that did not, indicating that this was unlikely.

## Conclusion

SEPs are a well-established first-line treatment for patients with IC and they are recommended by NICE guidelines. Despite this, many patients still do not have access to an exercise programme, and clinicians do not feel that they have support from funding bodies and commissioning groups to develop them. There is an urgent need for funding, development and delivery of supervised exercise services in the United Kingdom. Furthermore, there is a need for a greater evidence base for home-based exercise services to aid delivery where a face-to-face service may be impractical.

## Supplemental Material

sj-pdf-1-vas-10.1177_17085381211035259 – Supplemental Material for Early and midterm outcomes of hybrid first line treatment in patients with chronic limb threatening ischemia Click here for additional data file. Supplemental Material, sj-pdf-1-vas-10.1177_17085381211035259 for Provision of exercise services in patients with peripheral artery disease in the United Kingdom by Amy E Harwood, Sean Pymer, Said Ibeggazene, Lee Ingle, Eddie Caldow and Stefan T Birkett in Vascular
