# Supplementary material for: Provision of exercise services in patients with peripheral artery disease in the United Kingdom
Source: Vascular. 2021 Aug 4;30(5):874–81. doi: 10.1177/17085381211035259 (PMC9485155; doi:10.1177/17085381211035259)
Supplement: sj-pdf-1-vas-10.1177_17085381211035259 – Supplemental Material for Early and midterm outcomes of hybrid first line treatment in patients with chronic limb threatening ischemia [file sj-pdf-1-vas-10.1177_17085381211035259.pdf]

5/5/20

Audit Questions:

***Depending on the respondents answer they will be directed to specific areas of the survey via the complex routing process online**

Page 2

1. What is your occupation?
   a. Vascular consultant
   b. Vascular registrar
   c. Nurse
   d. Physiotherapist
   e. Exercise professional
   f. Occupational therapist
   g. Other

2. What city / town are you located in?

3. Please provide the name of your hospital trust

Page 3

1. Do you have access to an exercise programme for patients with peripheral arterial disease?** This is a routing question
   a. Yes
   b. No
   c. I don’t know

Page 4

1. Where is your exercise programme located?
   a. Hospital
   b. Community leisure facility
   c. Spoke service
   d. Other (free text)

2. How is the exercise programme funded?
   a. Research funded only
   b. NHS funded only
   c. Both
   d. I don’t know
   e. Other (free text)

3. Do you know how many people were eligible or completed exercise programmes?** This is a routing question
    a. Yes
    b. No
    c. I don't know

Page 5

1. Please provide the number of eligible patients referred to the exercise programme in the last year. If unsure, please write I don't know (free text).

2. Please provide the number of patients who started the exercise programme in the last year. If unsure, please write, I don't know (free text).

3. Please provide the number of patients who completed the programme. If unsure, please write I don't know (free text).

Page 6

1. Who is the clinical lead for the exercise programme?
    a. Vascular consultant
    b. Nurse
    c. Physiotherapist
    d. Occupational therapist
    e. Exercise professional
    f. Other (free text)

2. Who is responsible for the day-to-day running of the programme (i.e. taking the exercise sessions)?
    a. Vascular nurse
    b. Physiotherapist
    c. Physiotherapy assistant
    d. Exercise professional
    e. Other (open text box)

3. Does anyone else assist in the day-to-day running of the programme?
    a. No
    b. Nurse
    c. Physiotherapist
    d. Physiotherapy assistant
    e. Exercise professional
    f. Other (free text)

4. Does the person responsible for the day-to-day running of the exercise programme have any specific exercise qualifications (select all that apply).
   a. No
   b. British Association for Cardiovascular Prevention and Rehabilitation (BACPR) Exercise Instructor
   c. British Association of Sport and Exercise Sciences (BASES) Accreditation
   d. BASES Certified Exercise Practitioner
   e. American College of Sport Medicine (ACSM) Clinical Exercise Physiologist
   f. Register of Exercise Professionals (REPS)
   g. I don't know
   h. Other (open text box)

5. Do you provide any formal education to patients prior to entering an exercise programme, outside of the routine clinic appointments? Please select all that apply.
   a. No
   b. We provide comprehensive education including; smoking cessation, lifestyle and behaviour advice, dietary guidance, information about the disease and how to manage it.
   c. Smoking cessation and exercise advice
   d. Smoking cessation
   e. Exercise advice
   f. Behaviour intervention
   g. Dietary guidance
   h. I don't know
   i. Other (free text)

6. Do you provide patients with recommendations for increasing their habitual activity levels such as home exercise programme planning? This must be in addition to supervised exercise.
   a. No
   b. Home-exercise booklet
   c. Pedometer
   d. Smart watch
   e. Mobile app
   f. Regular phone calls
   g. Other

7. Do you assess patients' cardiovascular risk factors prior to entering an exercise programme?** This is a routing question
   a. Yes
   b. No
   c. I don't know

<u>Page 7</u>

1. What risk factors do you assess? Please select all that apply
    a. Baseline blood pressure
    b. Height
    c. Weight
    d. BMI
    e. Waist circumference
    f. Smoking status
    g. Cholesterol level
    h. If the patient is on best medical therapy
    i. Other (free text)

Page 8

1. Do patients undergo baseline testing prior to entering an exercise programme?** This is a routing question
    a. Yes
    b. No
    c. I don't know

Page 9

1. What baseline measures do you take from patients? Please select all that apply.
    a. ABPI at rest
    b. ABPI after exercise
    c. Pain-free and maximum walking distance via a graded treadmill test
    d. Pain-free and maximum walking distance via a constant load treadmill test
    e. Incremental shuttle walk test
    f. Six-minute walking distance
    g. CPET
    h. Spirometry
    i. Strength assessment (such as a one repetition max)
    j. Other (free text)

Page 10

1. Do you undertake any quality of life assessment on patients prior to entering the exercise programme? Please select all that apply
    a. We don't do any quality of life assessment
    b. Walking impairment questionnaire
    c. Short-Form 36
    d. VascuQol
    e. ED-5Q
    f. Edinburgh claudication questionnaire

g. Hospital Anxiety and Depression Scale
h. I don't know
i. Other (free text)

2. Following baseline testing, do the instructors follow any specific guidelines for their exercise prescription, if so what?
    a. Patients "self-prescribe"
    b. Prescribed on the claudication pain scale (e.g. 0-4 or 1-5).
    c. Prescribed as a % of HRmax
    d. Prescribed as a % of HRreserve
    e. Prescribed on the rating of perceived exertion (RPE)
    f. Other (free text)

3. How long does your exercise programme last (in weeks)? If unsure, please write "I don't know"

4. How many times does your patient attend supervised classes per week? If unsure, please write "I don't know"

5. How long do the individual sessions last (in minutes)? If unsure, please write "I don't know"

6. How do you record session attendance? If unsure, please write "I don't know"

7. Is your programme a group-based programme and if so, how many people on average take part in the exercise programme?** This is a routing question
    a. Yes
    b. No
    c. I don't' know

<u>Page 11</u>

1. How many patients on average take in the exercise programme per week?
    a. Less than 5
    b. 5 – 10
    c. 10 – 15
    d. More than 15
    e. I don't know
    f. Other

2. Is the primary condition for your group-based programme peripheral arterial disease? This means the class is exclusively provided for vascular patients as a vascular service.
    a. Yes
    b. We have PAD patients in a cardiac rehabilitation setting

c. We have PAD patients in a pulmonary rehabilitation setting
d. We have PAD patients in a multi-morbidity rehabilitation setting
e. Other (free text

Page 12

1. What type of exercises do you undertake as part of your programme?
    a. Walking on a treadmill only
    b. Walking in a gym facility only
    c. Circuit format which includes a component of walking
    d. Circuit training only
    e. Resistance training only
    f. A combination of walking and resistance training
    g. Upper-body cycling exercise
    h. High-intensity interval training
    i. I don't know
    j. Other (free text)

2. When a patient completes an exercise programme do you undertake follow-up testing?
    a. ABPI at rest
    b. ABPI after an exercise test
    c. Pain-free and maximum walking distance via a graded treadmill test
    d. Pain-free and maximum walking distance via a constant load treadmill test
    e. Incremental shuttle walk test
    f. Six-minute walk test
    g. CPET
    h. Spirometry
    i. Strength measures (such as one repetition maximum)
    j. We don't undertake follow up
    k. I don't know
    l. Other (free text)

3. When patients don't want to undertake an exercise programme what barriers are cited? Please select all that apply
    a. Time (getting to the programme)
    b. Time (not enough personal time)
    c. No transport / inability to access public transport
    d. Not financially able to attend the programme
    e. Too much pain when walking
    f. Work commitments
    g. Other (free text)

4. What resources would you use to make changes to your exercise programme? Please select all that apply

a. National guidance such as NICE
b. Charity information pages, such as the Circulation Foundation
c. Latest research articles
d. Sport and exercise science statements
e. Word of mouth / expert opinion
f. Other (free text)

5. What would help you improve your exercise programme? Please select the most important factor to you.**This is a routing question
    a. Additional staff members with expertise
    b. More funding
    c. Access to exercise facilities
    d. More resources such as equipment
    e. More support from senior staff members
    f. Better knowledge
    g. No improvement needed
    h. Other (free text)

Page 13 * Directed from page 3 question

1. What information do you provide to patients if you don't have a specific exercise programme? Please select all that apply
    a. Basic walking advice
    b. Structured exercise advice (with specific prescription)
    c. Smoking cessation guidance
    d. Home exercise booklet
    e. Dietary guidance
    f. Nothing
    g. I don't know
    h. Other (free text)

2. What do you think are the barriers to implementing an exercise programme in your trust? Please select all that apply
    a. Funding
    b. Expertise
    c. Equipment
    d. Facilities to deliver the sessions
    e. Staffing
    f. Other (free text)

3. Where would you look for information about starting an exercise programme? Please select all that apply
    a. Academic journals / papers
    b. Charity websites such as the Circulation Foundation

   c. Clinical guidance such as NICE
   d. Expert knowledge / opinion
   e. Other (free text)

4. What would help you implement an exercise programme?

Page 14 – all respondents to answer

1. Do you think taking part in an exercise programme is important to patients?
   a. Not at all important
   b. Slightly important
   c. Moderately important
   d. Very important
   e. Extremely important

2. Do you think exercise programmes are valued and prioritised by clinicians in your centre?
   a. Not at all important
   b. Slightly important
   c. Moderately important
   d. Very important
   e. Extremely important

3. Do you think exercise programmes are valued and prioritised by commissioning groups and funding bodies?
   a. Not at all important
   b. Slightly important
   c. Moderately important
   d. Very important
   e. Extremely important
